# Unravelling the low-temperature metastable state in perovskite solar cells by noise spectroscopy

**DOI:** 10.1038/srep34675

**Published:** 2016-10-05

**Authors:** C. Barone, F. Lang, C. Mauro, G. Landi, J. Rappich, N. H. Nickel, B. Rech, S. Pagano, H. C. Neitzert

**Affiliations:** 1 Dipartimento di Fisica “E.R. Caianiello” and CNR-SPIN Salerno, Università di Salerno, I-84084 Fisciano, Salerno, Italy; 2 Helmholtz-Zentrum Berlin für Materialien und Energie GmbH, Institut für Silizium Photovoltaik, Kekuléstr. 5, 12489 Berlin, Germany; 3 Dipartimento di Ingegneria Industriale, Università di Salerno, I-84084 Fisciano, Salerno, Italy

## Abstract

The hybrid perovskite methylammonium lead iodide CH_3_NH_3_PbI_3_ recently revealed its potential for the manufacturing of low-cost and efficient photovoltaic cells. However, many questions remain unanswered regarding the physics of the charge carrier conduction. In this respect, it is known that two structural phase transitions, occurring at temperatures near 160 and 310 K, could profoundly change the electronic properties of the photovoltaic material, but, up to now, a clear experimental evidence has not been reported. In order to shed light on this topic, the low-temperature phase transition of perovskite solar cells has been thoroughly investigated by using electric noise spectroscopy. Here it is shown that the dynamics of fluctuations detect the existence of a metastable state in a crossover region between the room-temperature tetragonal and the low-temperature orthorhombic phases of the perovskite compound. Besides the presence of a noise peak at this transition, a saturation of the fluctuation amplitudes is observed induced by the external DC current or, equivalently, by light exposure. This noise saturation effect is independent on temperature, and may represent an important aspect to consider for a detailed explanation of the mechanisms of operation in perovskite solar cells.

A recent breakthrough in dye-sensitized solar cells has been the introduction of organic-inorganic hybrid perovskite materials, such as CH_3_NH_3_PbI_3_, to improve the power conversion efficiency^1^. Although not very efficient at the beginning, perovskite solar cells have quickly become very promising low-cost alternatives to traditional semiconductors, due to a regular achievement of 15% in efficiency with an NREL certified current record exceeding 22%^2^.

The use of perovskites in photovoltaics has attracted the scientific community, due to their peculiar chemical and physical properties, associated with a simple synthesis. In particular, it has been found that perovskites have bandgap energies in the near-infrared close to the Shockley-Queisser ideal limit^1^, elevated charge carrier mobilities, diffusion lengths on the order of micrometers^3^, and long charge carrier lifetimes^4^. All these characteristics, added to a certified stability under high proton irradiation, are useful for the development of a new and competitive photovoltaic technology, also functional for space applications^5^. In addition, hybrid perovskites are an ideal candidate for tandem solar cells with silicon, due to the low sub-bandgap absorption and the high bandgap energies. Various innovative concepts already showed the potential to outperform traditional single junction silicon solar cells^6^,^7^,^8^,^9^.

However, a clear picture of the electrical transport mechanisms in perovskites has not been achieved. Indeed, while there is a wide scientific literature on the photophysics of these materials in the room and high temperature regimes^10^,^11^,^12^,^13^,^14^,^15^, the understanding of how the lowering of temperature affects the charge carrier dynamics remains, instead, scarce. As is quoted by Loi and Hummelen^12^: “*it is now time to investigate all the physical properties that make hybrid perovskites so promising for solar energy conversion*”. In this respect, the carrier kinetic processes after optical excitation have been investigated^3^, and theoretical calculations have been performed by using experimental findings from: temperature-dependent absorption spectra, millimeter-wave spectroscopy, low-frequency dielectric measurements, and NMR data related to the motion of molecular cations^16^. Photoluminescence properties, under one-photon and two-photon excitation, have been also studied in CH_3_NH_3_PbBr_3_ microdisks, giving a more comprehensive understanding of the nonlinear effect of organic-inorganic perovskite single crystals^17^.

Among the available non-destructive characterization techniques, noise spectroscopy has demonstrated its effectiveness in the investigation of advanced photovoltaic and innovative materials^18^,^19^,^20^,^21^. Preliminary characterizations have also been performed in mesoscopic perovskite photodiodes^22^. Moreover, by studying the frequency and temperature dependencies of the 1/f spectral noise density, changes in the structural properties of nickel-titanium binary alloys^23^ and of iron-based superconductors^24^,^25^ have been revealed. In the case of perovskite solar cell devices, where a phase transition from a tetragonal to an orthorhombic structure has been observed near 160 K^26^, noise measurements can be a very useful experimental technique, considering that no clear signature of such a transition is evident in the cell DC properties.

In this work, a detailed analysis of the fluctuation processes has been performed in methylammonium lead iodide (CH_3_NH_3_PbI_3_) samples by varying temperature, bias current, and illumination level. The reported results shine light on the role played by excitons in the photoexcited carrier dynamics, and on the temperature dependence of localization effects, including phonon coupling and charge carrier trapping^27^,^28^. These phenomena need to be clarified, because of their potential to influence the efficiency of charge transport within the perovskite film.

## Results

### Structural and photoelectric properties

**Figure 1:**
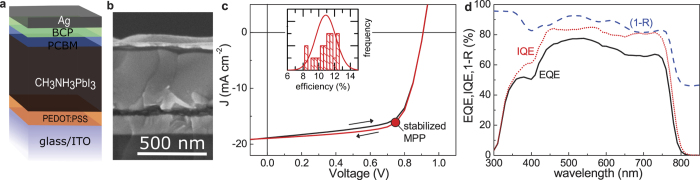
Perovskite solar cell characteristics at room temperature. (**a**) Sketch of the used inverted perovskite solar cell structure. (**b**) Cross sectional scanning electron micrograph. (**c**) Current density-voltage characteristics of the best performing device. Black and red lines refer to performed forward and backward voltage scans, respectively. The result of a maximum power point tracking after stabilization verifies the power conversion efficiency of 12% (red dot). The inset shows a histogram of the measured efficiency of 20 processed devices. (**d**) The wavelength-dependent external (EQE) and internal quantum efficiency (IQE) plotted as black and red lines. The blue dashed line refers to the measured reflection *R* as (1 − *R*).

Hybrid perovskites are an efficient light harvester within mesoscopic solar cells^29^. However, when using such a mesoscopic TiO_2_ scaffold, good charge carrier transport properties are unnecessary. Performance, instead, is related to the interfacial properties^13^. Today’s best performing devices, on the other hand, rely on thicker perovskite layers^30^,^31^, as it is also the case of the photovoltaic devices studied here. The understanding of the carrier transport mechanisms, therefore, becomes important^10^,^11^. Figure 1a shows a typical device scheme of an inverted perovskite solar cell, consisting of the layer stack glass/ITO/PEDOT:PSS/CH_3_NH_3_PbI_3_/PC_61_BM/BCP/Ag. Figure 1b shows a cross sectional scanning electron micrograph. The perovskite absorber has a thickness of around 430 nm. Identically processed devices revealed an average efficiency about 11% at room temperature and simulated AM 1.5G illumination. The current density-voltage (*J*-*V*) characteristics of the best performing device, with an efficiency of 12.1%, is depicted in Fig. 1c. In contrast to perovskite solar cells based on TiO_2_^32^, the inverted device is hysteresis free^33^, as evident from the shown *J*-*V* sweeps in forward and backward direction. A power point tracking algorithm additionally verifies an efficiency of 12.04% after stabilization, as indicated by the red dot (see also Supplementary Fig. S1). The external (EQE) and internal quantum efficiency (IQE) are depicted in Fig. 1d. The integrated short-circuit current density amounts to 17.4 mA cm^−2^, and, therefore, matched the current obtained under AM 1.5G illumination.

**Figure 2:**
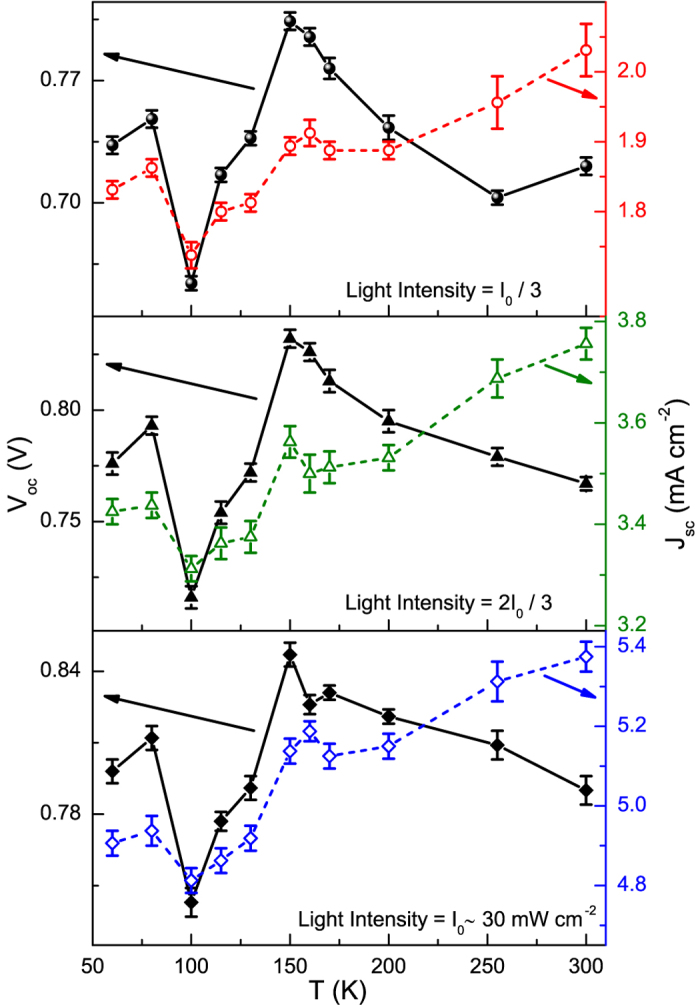
Optoelectronic properties from room temperature down to 60 K. The temperature dependencies of the open-circuit voltage *V*_*oc*_ (left axis) and of the short-circuit current density *J*_*sc*_ (right axis) are shown at three distinct illumination levels, supplied with a cool white LED as light source.

In addition to the typical standard test conditions at room temperature, perovskite solar cells were probed as a function of temperature from 300 K down to 60 K. This time, a commercial white Light Emitting Diode (LED) was used as light source (see Methods section for details). In Fig. 2, the temperature dependencies of the open-circuit voltage *V*_*oc*_ (left axis) and of the short-circuit current density *J*_*sc*_ (right axis) are shown at three different illumination levels. A change in the behaviour of *V*_*oc*_ (black closed symbols), in a temperature range between 150 and 100 K, is clearly evident, while a less pronounced effect is found for *J*_*sc*_ (coloured open symbols) independently on the light intensity. This result suggests the occurrence of a modification in the electrical properties of the photovoltaic device. However, no specific information can be extracted on the typology of the observed phenomenon. The question is whether the structural transition from a tetragonal to an orthorhombic phase (occurring around 160 K) plays any role in the low-temperature conduction regime of perovskite photovoltaic devices. In order to shed light on this aspect, a more sensitive investigation technique is needed.

### Electric noise spectroscopy

**Figure 3:**
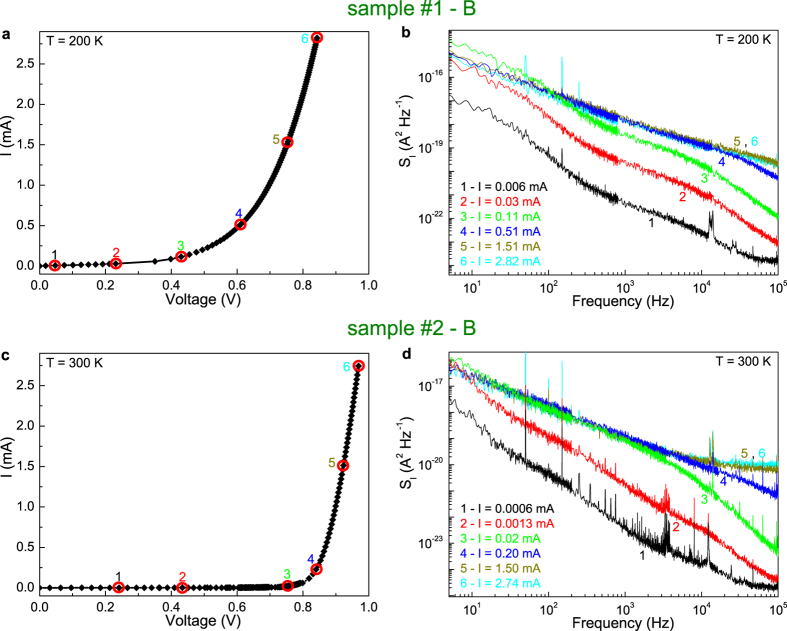
Noise spectra at different bias points. (**a**,**c**) Dark *I*-*V* characteristics at temperatures of 200 and 300 K are shown for two perovskite solar cells: sample #1 device B and sample #2 device B, respectively. (**b**,**d**) The frequency dependence of the current-spectral density *S*_*I*_ is shown for the two considered photovoltaic devices. The bias points, at which noise spectra have been acquired, are numbered from 1 (lowest current value used) to 6 (highest current value used).

Electric noise analysis is usually made by measuring the spectral density of voltage fluctuations *S*_*V*_ in a DC current biased sample. In semiconducting devices, the main noise source is due to charge number fluctuations, therefore, a more representative quantity for the noise spectral density is 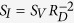, where *R*_*D*_ is the differential resistance at the DC bias point. In Fig. 3a,c, the *I*-*V* characteristics are shown at two temperatures (200 and 300 K, respectively) for two distinct samples, named as sample #1 (“thin”) and sample #2 (“thick”), whose structural difference can be clearly observed in the scanning electron micrographs of Supplementary Fig. S2. Despite the shape of dark *I*-*V* curves looks different, the photoelectric parameters of the investigated devices are similar (see the values reported in Supplementary Fig. S3), and a general trend can be also found in the current-noise spectra. This is evident in Fig. 3b,d, where the low-frequency dependence of *S*_*I*_ is shown for the corresponding bias points of Fig. 3a,c. More in details, *S*_*I*_ increases with the applied bias current until the occurrence of a saturation for values higher than 0.2 mA (see the blue spectral traces 4 in Fig. 3b,d). When the electric noise is saturated, a pure 1/f component is visible, as shown by curves 5 and 6 in Fig. 3b,d. Conversely, in the low bias current limit, *S*_*I*_ is characterized by the presence of two cut-off frequencies, each one specific of a change from a 1/f to a 1/f^3^ dependence of the spectrum (spectral traces 1–3 of Fig. 3b,d). In particular, the highest cut-off frequency can be defined in terms of a time constant which describes the recombination phenomena associated to the defect states^18^. From noise spectroscopy, this time constant is estimated to assume temperature-dependent values between 4 and 10 *μ*s, giving the experimental quantification of the rate constant for the physical process that produces the electric noise in perovskite solar cells. It is clear, however, that there is a peculiar feature of the charge carrier fluctuation mechanisms, indicating a crossover between distinct noise regimes. This behaviour is confirmed on several devices, and at different temperatures, as shown by Supplementary Fig. S4.

**Figure 4:**
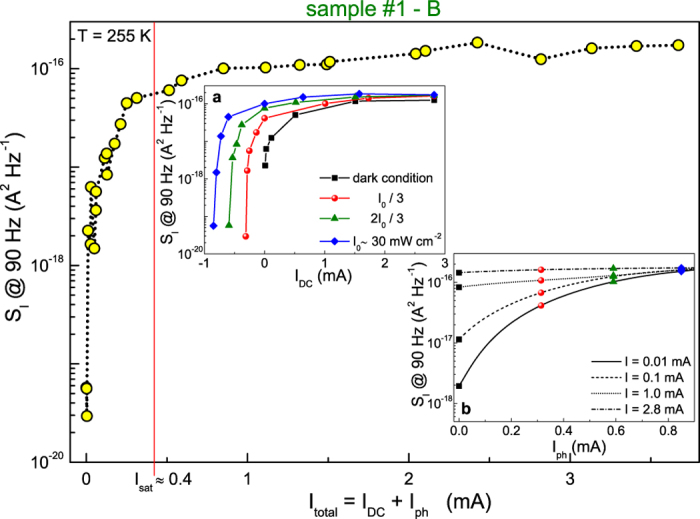
Noise current dependence in dark and under light exposure. The spectral density *S*_*I*_, at a frequency of 90 Hz, is shown as a function of the total current for sample #1 device B at a fixed temperature of 255 K. A saturation of the noise amplitude is observed by varying, independently, the DC applied bias *I*_*DC*_ (inset **a**) and the photogenerated current *I*_*ph*_ (inset **b**). By considering the sum *I*_*DC*_ + *I*_*ph*_, the noise saturation effect is estimated to occur near 0.4 mA.

The existence of a DC bias-induced noise saturation has been observed in dark condition, and is found also under light exposure. This is clearly visible in the inset a and inset b of Fig. 4, where the current noise computed at a reference frequency of 90 Hz is shown as a function of the DC bias current *I*_*DC*_ and of the photogenerated current *I*_*ph*_, at a temperature of 255 K. By plotting the total current (*I*_*DC*_ + *I*_*ph*_) dependence of the noise amplitude, all the experimental data distribute on a single curve (see Fig. 4). This confirms that the origin of the measured electric noise is due to the fluctuation of total charge carriers present in the device, either photogenerated or injected by the bias leads. In addition, a saturation of the noise amplitude is observed for currents above 0.4 mA, a value that is sample- and temperature-dependent. Although space-charge-limited current measurements may be very useful for a better understanding and, therefore, are currently in progress, an explanation of this behaviour can be given using the well-known excitation-trapping fluctuation model^34^. In this framework, the noise process derives from the assumption that free carriers are randomly trapped and released by centers with different times. The generation of carriers, induced by DC bias or by photocurrent, increases the trap filling until a noise saturation occurs, when all the traps are active.

**Figure 5:**
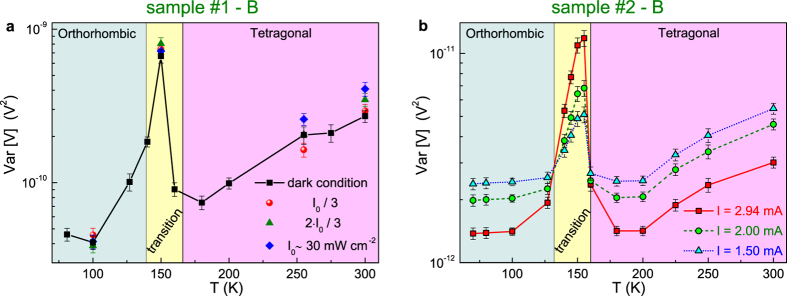
Temperature dependence of the noise level. (**a**) The variance of the measured voltage fluctuations *Var*[*V*], at a fixed bias current value of 2.9 mA, is shown as a function of temperature for sample #1 device B. The black squares refer to data taken in dark condition, while red circles, green triangles, and blue diamonds correspond to three different illumination levels reported in the legend. (**b**) The temperature behaviour of the voltage-noise amplitude is shown for sample #2 device B, by varying the applied bias current. In all the cases considered, the presence of a noise peak is clearly visible between 150 and 160 K.

An useful quantity to estimate the overall generated noise is the variance of voltage fluctuations *Var*[*V*], obtained by computing the integral of *S*_*V*_ in the frequency domain between [*f*_*min*_, *f*_*max*_] = [1, 100000] Hz (the experimental bandwidth). In Fig. 5 the temperature dependence of *Var*[*V*], measured at large bias current (that is in the region of full trap activation), is shown for the investigated samples, characterized by different perovskite grains sizes and cristallinity. The reported values differ by two orders of magnitude, between sample #1 and sample #2, when similar bias currents are applied (2.9 mA for sample #1 and 2.94 mA for sample #2). By excluding a parasitic effect of the involved interfaces, which are identically processed, the measured noise fluctuations are dominated by a defect related trapping/detrapping mechanism within the bulk perovskite absorber. Therefore, the lower *Var*[*V*] of sample #2 indicates a lower trap density in the device of large grain size. The finding is consistent with the reported increase of the electron-hole diffusion length with increasing grain size^10^,^35^,^36^. The data of Fig. 5a, acquired both in dark and at different illumination intensity, reveal the presence of a noise peak at temperatures between 150 and 160 K. This feature is independent on the presence of photogenerated carriers, however it shows a clear dependence on the bias current, as visible in Fig. 5b. Moreover, above 160 K and below 130 K, the noise level decreases by increasing the current. Surprisingly, the opposite behaviour is found in the temperature region around the noise peak (see Fig. 5b). It is conceivable that the observed noise peak is related to the perovskite phase transition occurring in the temperature range near 160 K. A detailed discussion on how this influences the fluctuation processes is given below, providing a better understanding on the physical properties of perovskite photovoltaic devices.

## Discussion

The repeatability and the intrinsic nature of the observed temperature noise behaviour have been verified by measuring the samples in cooling and heating modes, in the range between 70 and 300 K. The data shown in Supplementary Fig. S5 give a clear indication that hysteretic effects are not present in the experimental results, and no significant difference characterizes the temperature direction change.

After the verification of the reproducibility of the noise peak found near 155 K, a detailed study of the voltage-noise amplitude has been done for different bias points. In order to explain the measured voltage-spectral densities, the overall noise can be expressed as the sum of two independent contributions


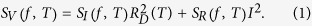


In equation (1), the first term represents a current-noise source that is always present and takes into account the fluctuations due to active trapping states in the photovoltaic device. As specified before, this is modeled by the well-known trapping/detrapping mechanism, which induces charge carrier fluctuations and, consequently, a current noise *S*_*I*_^34^. The second term of equation (1) represents a voltage-noise source that, instead, describes a resistance network fluctuation mechanism *S*_*R*_, occurring in the perovskite phase-transition region where metastable states between local resistive states are established. While conventional silicon-based solar cells have already shown the presence of trapping-detrapping related processes^37^, resistive-induced fluctuators are usually found in random network systems^38^ but are unexpected in photovoltaic materials.

**Figure 6:**
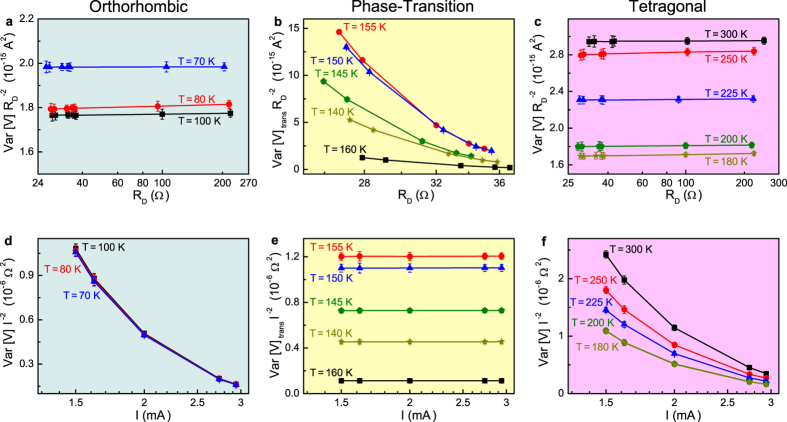
Behaviour of the noise amplitude in different temperature regions. (**a**,**c**) For sample #2 device B, the normalized noise amplitude 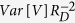 is shown by varying the value of the differential resistance. (**d**,**f**) The bias current dependence of the normalized noise *Var*[*V*]*I*^−2^ is also shown for the same photovoltaic device. (**b**,**e**) The similar behaviour found for the tetragonal (*T* > 160 K) and the orthorhombic (*T* < 130 K) phases is no more observed in the transition region between 160 and 130 K, where the excess noise contribution *Var*[*V*]_*trans*_ has been computed and reported.

In Fig. 6a,c, the measured voltage-spectral density amplitudes are shown in the low (orthorhombic) and high (tetragonal) temperature regions, respectively. The experimental results indicate a direct proportionality of the noise level with the differential resistance squared, and are consistent with the presence of a current-noise source alone (first term of equation (1)). This noise contribution increases with temperature above 200 K, while, below 200 K, is substantially constant. However, in the region around 160 K, the experimental behaviour is completely different and is characterized by the appearance of an excess noise component, which can be extracted by subtracting from the measured noise level the constant current-noise term. The values of this excess noise contribution, shown in Fig. 6b,e as *Var*[*V*]_*trans*_, seem to scale well with the applied bias squared *I*^2^ and, conversely, have no relation with 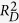.

The unexpected additional noise observed near 160 K can be explained by considering that, in this temperature region, the structural reorganization of the perovskite material produces a random distribution of microscopic phases, to which a random resistor network can be associated. The resistors fluctuate randomly between the values characteristic of the two perovskite phases, giving rise to a 1/f noise with a quadratic bias current dependence. This is exactly what is shown in Fig. 6e, where the excess noise level scaled with *I*^2^ exhibits a constant value, different for the specific temperatures investigated. The highest value is found at 155 K, indicating this as the structural transition temperature of the perovskite compound.

Figure 6d,f show that the quadratic bias scaling is not present outside the phase-transition region, confirming the proposed model. As a consequence, from the noise point of view, the low-temperature crossover between the tetragonal and orthorhombic phases can be clearly identified by the change from a trapping/detrapping to a resistance fluctuation mechanism. The structural reorganization of the microscopic metastable states, established in the transition region, may be characterized by long-term relaxation processes, which are probably connected with the two turning points (around 150 and 100 K) observed in the *V*_*oc*_ temperature dependence (see Fig. 2 for details). More information on important electronic parameters of the solar cell defect states can be extracted by using a current-noise theoretical interpretation^37^, whose applicability to the case of perovskite compounds is currently in progress.

## Conclusion

Hybrid perovskites are an ideal absorber for solar cells, but show various structural transitions upon temperature. For example, the CH_3_NH_3_PbI_3_ compound, here studied, changes from a tetragonal to an orthorhombic structure at low temperature. As a result the optoelectronic parameters, such as open-circuit voltage and short-circuit current density, present signs of irregular behaviour between 150 and 100 K. The DC analysis alone, however, is not able to provide clear indications on what is really happening. In this respect, it is possible to obtain much more information by the electric noise spectroscopy. In particular, the appearance of a noise level peak, independently on the illumination condition, allows an estimation of a temperature region where the nature of fluctuation mechanisms changes. Above 160 K (in the tetragonal phase) and below 130 K (in the orthorhombic phase), the electric noise is generally produced by a trapping/detrapping process. Resistance fluctuations, instead, are dominant in the phase-transition region, near the noise level peak. When the active traps are filled by the charge carrier generation, the trapping noise contribution saturates. Conversely, the amplitude of resistance fluctuations increases with the squared bias current, revealing the existence of a metastable state in the crossover between the tetragonal and orthorhombic structures of the perovskite compound. This metastable state may be attributed to a random distribution of microscopic phases. Therefore, in simple and complex mixed perovskite solar cells, where structural reorganizations induced by light, time, and temperature seem to be very relevant, a non-destructive and simple experimental technique, such as noise spectroscopy, can play an important role for the understanding of the physics of such devices.

## Methods

### Solar cells preparation

Perovskite solar cells were prepared following the layer sequence glass/ITO/PEDOT:PSS/CH_3_NH_3_PbI_3_/PC_61_BM/BCP/Ag. Therefore, patterned glass/ITO substrates were cleaned with acetone, detergent/H_2_O, H_2_O, and isopropanol. After a final plasma cleaning, a 60 nm thick PEDOT:PSS layer (Heraeus PH 4083) was prepared by spin-coating at 3000 rpm for 30 s. The obtained films were annealed at 150 °C for 20 min, before being transferred to inert atmosphere. A stoichiometric CH_3_NH_3_PbI_3_ precursor solution, containing either 0.8 M (thin sample) or 1.1 M (thick sample) of PbI_2_ (Sigma-Aldrich) and CH_3_NH_3_I (synthesized from CH_3_NH_2_ and HI^26^, Sigma-Aldrich) in the mixed solvent *γ*-butyrolactone (GBL) and dimethyl sulfoxide (DMSO) with volume ratio 70/30 (v.%/v.%), was prepared under stirring at 60 °C for 12 h. The CH_3_NH_3_PbI_3_ absorber was spin-coated at 1000 rpm for 10 s, 2000 rpm for 20 s, and 5000 rpm for 20 s. In the final spin-coating stage, 150 *μ*l toluene were dripped onto the sample, according to Jeon *et al.*^39^. After crystallization at 100 °C for 10 min, a smooth CH_3_NH_3_PbI_3_ layer was obtained. The electron selective contact was formed by spin-coating a 50 nm thick PC_61_BM layer (Lumtec, 99.5%, 20 mg ml^−1^ in chlorobenzene) at 2500 rpm for 60 s. After annealing at 100 °C for 10 min, a thin layer of bathocuproine (BCP, 0.5 mg ml^−1^ in ethanol, Sigma-Aldrich) was spin-coated at 4000 rpm for 45 s. The devices were completed by thermal evaporation of 100 nm Ag at a base pressure of 10^−7^ mbar through a shadow mask. The overlap of the patterned ITO and the metal contact defined the active area of prepared devices to 0.16 cm^2^. For noise measurements, samples were encapsulated using a cover glass and two-component epoxy.

### Room temperature DC and structural measurements

The room temperature characterization of perovskite solar cells was performed under simulated AM 1.5G light provided by a “Steuernagel Lichttechnik GmBH”, or Newport LCS-100 class ABB sun simulator, both calibrated using an ISE certified Si reference solar cell. Current density-voltage (*J*-*V*) scans were performed with a Keithley 2400 in forward and reverse directions, using a sweep speed of 85 mV s^−1^. Before measurement, the devices were light soaked in inert atmosphere for 30 min. The external quantum efficiency was measured without bias voltage and bias illumination, using a calibrated “Oriel QEPVSI-b” setup. Reflection was measured with a PerkinElmer UV-vis spectrometer, equipped with an integrating Ulbricht sphere and calibrated from an ISE certified white standard. Cross sectional scanning electron micrographs (SEM) were recorded with a Hitachi S-4100 at 5 kV acceleration voltage.

### Low temperature DC and noise measurements

All the measurements, performed by varying the temperature, were carried out in a closed-cycle refrigerator, operating in the 8- to 300-K range. A computer-controlled feedback loop realized the temperature stabilization. The achieved stability, better than 1 K, was sufficient to record stable spectral data at all investigated temperatures, measured with a Cernox resistor thermometer in contact with the sample holder. The photovoltaic devices were biased with a low-noise Keithley DC current source. The DC voltage drop was measured with a digital multimeter, while the AC voltage signal, amplified with a low-noise PAR5113 preamplifier, was analyzed with a dynamic signal analyzer HP35670A. The presence of spurious components in the measured spectral traces was removed by resorting to a specific procedure, based on a sequence of four-probe and two-probe noise measurements^40^, useful to eliminate unwanted contact noise contributions^41^. A commercial cool white Light Emitting Diode (LED) was used as light source, for noise investigations. This choice of adopting a LED has been done in order to have a low-noise source and to discriminate intrinsic photoinduced mechanisms from extrinsic ones, potentially related to the complex electronics of the solar simulator.

## Additional Information

**How to cite this article**: Barone, C. *et al.* Unravelling the low-temperature metastable state in perovskite solar cells by noise spectroscopy. *Sci. Rep.* **6**, 34675; doi: 10.1038/srep34675 (2016).

## Supplementary Material

Supplementary Information
