# Supplementary material for: Unravelling the low-temperature metastable state in perovskite solar cells by noise spectroscopy
Source: Sci Rep. 2016 Oct 5;6:34675. doi: 10.1038/srep34675 (PMC5050427; doi:10.1038/srep34675)
Supplement: Supplementary Information [file srep34675-s1.pdf]

Supplementary Information for "Unravelling the low-temperature metastable state in perovskite solar cells by noise spectroscopy"

C. Barone[1],* F. Lang[2], C. Mauro[1], G. Landi[3], J. Rappich[2],
N. H. Nickel[2], B. Rech[2], S. Pagano[1], and H. C. Neitzert[3]

[1]*Dipartimento di Fisica "E.R. Caianiello" and CNR-SPIN Salerno, Università di Salerno, I-84084 Fisciano, Salerno, Italy*

[2]*Institut für Silizium Photovoltaik, Helmholtz-Zentrum Berlin für Materialien und Energie GmbH, Kekuléstr. 5, 12489 Berlin, Germany*

[3]*Dipartimento di Ingegneria Industriale, Università di Salerno, I-84084 Fisciano, Salerno, Italy*

*Electronic address: cbarone@unisa.it

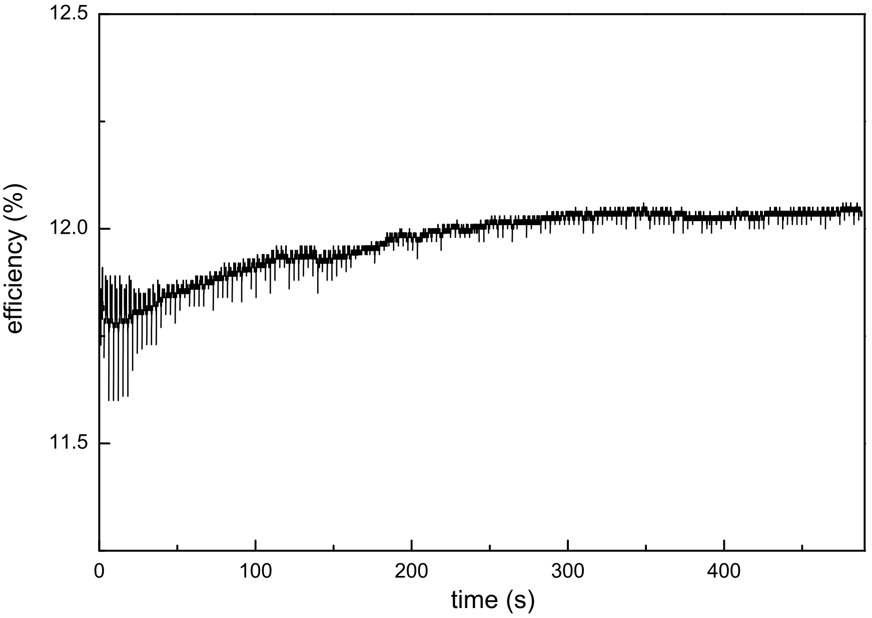

Fig. S1: **Efficiency from stabilized MPP.** Perovskite solar cell efficiency has been additionally evaluated from maximum power point (MPP) tracking versus time. For the best performing device, after stabilization a value of 12.04%, matching the result from J-V characteristics, is obtained.

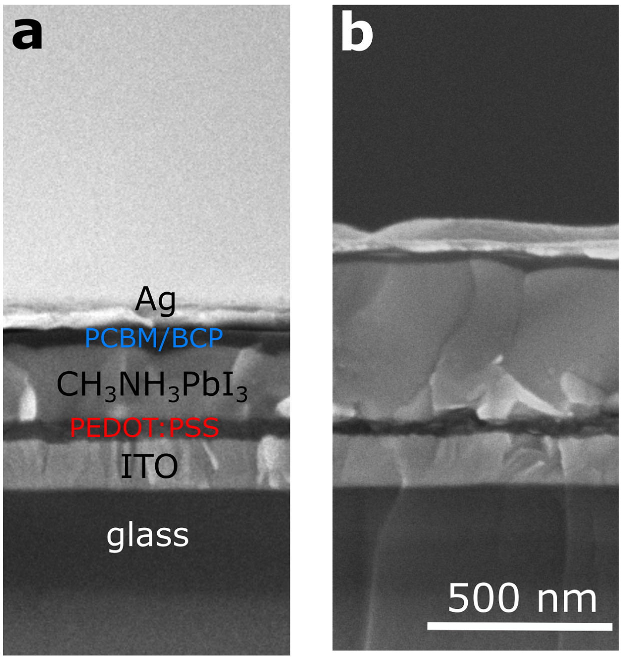

Fig. S2: **Cross sectional scanning electron micrographs of the investigated samples.** (**a**) The 210 nm "thin" device (sample #1) is shown. (**b**) The 430 nm "thick" device (sample #2) is shown.

**sample #1**												
Voltage	A_for	A_back	B_for	B_back	C_for	C_back	D_for	D_back	E_for	E_back	F_for	F_back
[V]	[mA/cm²]	[mA/cm²]	[mA/cm²]	[mA/cm²]	[mA/cm²]	[mA/cm²]	[mA/cm²]	[mA/cm²]	[mA/cm²]	[mA/cm²]	[mA/cm²]	[mA/cm²]
J_sc [mA/cm²]:	-17.93	-18.23	-18.23	-18.28	-18.60	-18.63	-17.91	-17.95	-18.38	-18.43	-18.54	-18.55
V_oc [V]:	0.68	0.68	0.69	0.69	0.68	0.68	0.67	0.67	0.68	0.68	0.68	0.68
Fill factor [%]:	65.49	67.39	64.28	64.84	66.37	66.78	66.44	68.43	57.17	58.16	68.89	68.50
Efficiency [%]:	9.28	8.44	8.11	8.20	8.34	8.40	9.28	8.44	7.19	7.34	8.76	8.65
**sample #2**												
Voltage	A_for	A_back	B_for	B_back	C_for	C_back	D_for	D_back	E_for	E_back	F_for	F_back
[V]	[mA/cm²]	[mA/cm²]	[mA/cm²]	[mA/cm²]	[mA/cm²]	[mA/cm²]	[mA/cm²]	[mA/cm²]	[mA/cm²]	[mA/cm²]	[mA/cm²]	[mA/cm²]
J_sc [mA/cm²]:	-18.17	-18.26	-18.11	-18.20	-18.33	-18.38	-18.25	-18.28	-17.26	-17.28	-17.44	-17.46
V_oc [V]:	0.89	0.89	0.90	0.89	0.89	0.89	0.90	0.89	0.89	0.89	0.89	0.89
Fill factor [%]:	64.85	66.81	66.86	68.88	67.71	69.24	67.54	69.25	66.28	68.17	65.79	67.47
Efficiency [%]:	10.52	10.89	10.85	11.21	11.09	11.35	11.04	11.32	10.17	10.47	10.21	10.47

Fig. S3: **Solar cells electrical parameters.** Short-circuit current density J_{sc}, open-circuit voltage V_{oc}, fill factor FF, and power conversion efficiency η are reported for investigated sample #1 and sample #2, each one formed by six independent devices (A$\cdots$F). The current-voltage measurements, in both forward and backward direction, have been performed at room temperature and at 1 sun illumination level.

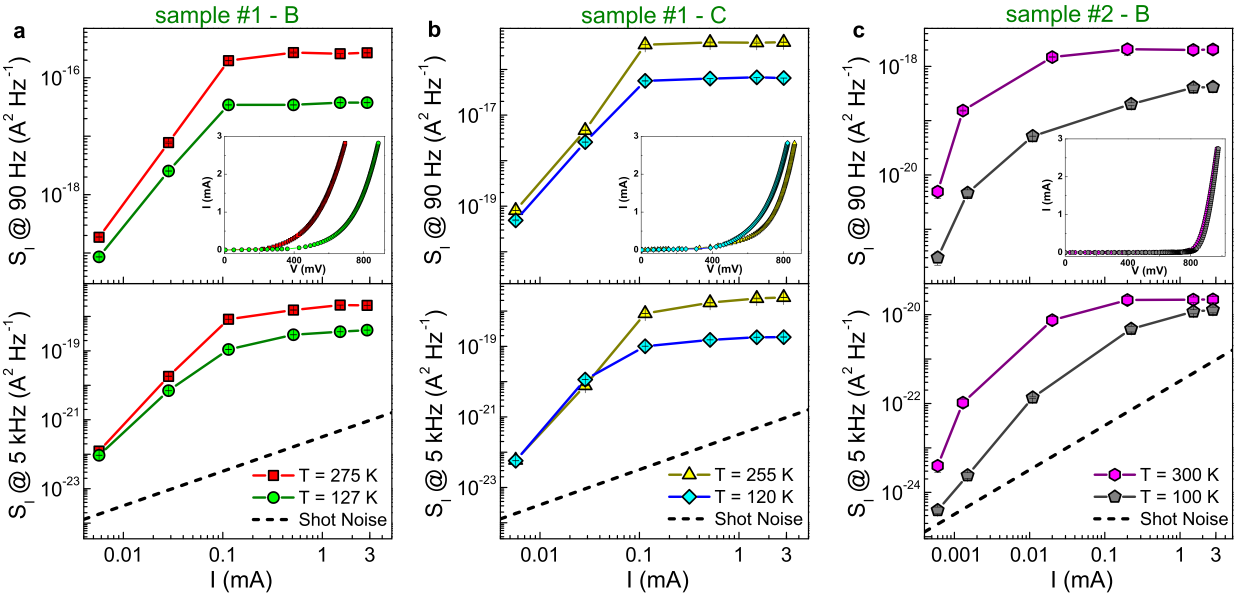

Fig. S4: **Dark noise current dependence.** At two reference frequencies of 90 Hz (upper panels) and of 5 kHz (lower panels), the current dependence of the dark noise is shown for different temperatures. The data refer to three solar cells: (**a**) sample #1 device B; (**b**) sample #1 device C; (**c**) sample #2 device B. Above a specific threshold current, sample- and temperature-dependent, a noise amplitude saturation effect is always observed.

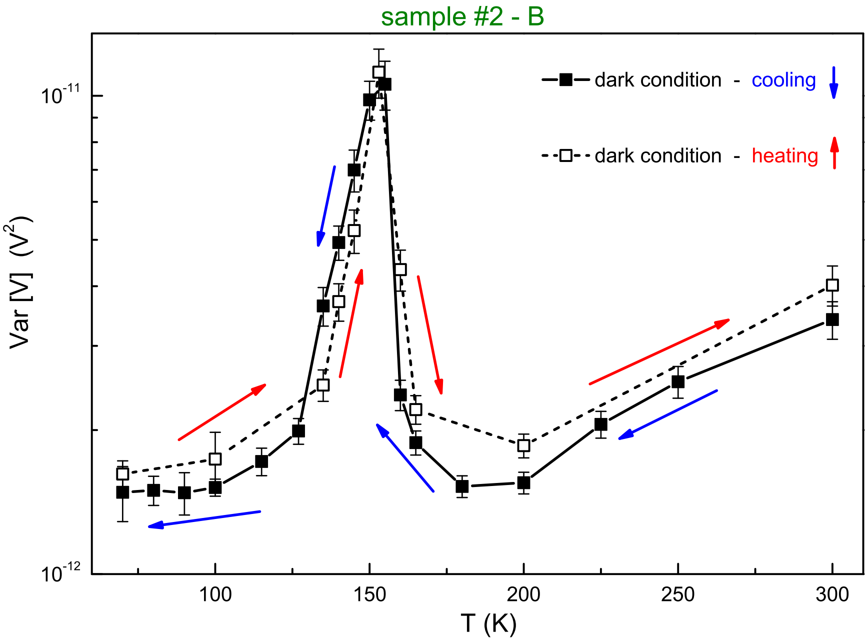

Fig. S5: **Temperature behaviour of the noise amplitude.** The variance of dark voltage fluctuations is shown for sample #2 device B by lowering the temperature down to 70 K (full squares and solid line), and by increasing the temperature up to 300 K (open squares and dashed line). The trend observed in the two distinct situations is very similar, with a difference between the measured values within the experimental errors. In particular, the structural transition, evidenced by the noise peak near 155 K, seems to be a reversible transformation, as suggested by the absence of hysteretic effects of the noise properties.
